# 

**Published:** 1878-09-20

**Authors:** 


### ORIGINAL AND SELECTED ARTICLES.

The Antiseptic Properties of Tar Water; by Thos. R. Wright........257
The Pith of Dried Cornstalk as a Uterine Tent; by W. T. Goldsmith, M. D., Atlanta........260
Tansy in Pruritus Vulvæ........262
Pond's American Sphygmograph; by Frank Woodbury, M.D........262
Scraping Spoon........264
The Treatment of Yellow Fever........265
Strangulated Hernia—Treatment by Hypodermic Injections of Morphia Hydrochloras; by G. H. Chapman, M.D........163
Magic Effects of Hypodermic Puncture of Morphia in Cases of Dysentery........270

### ABSTRACTS AND GLEANINGS.

Malarial and Yellow Fever, 271: The Cpinm Habit. 273. Chronic Intermittents, 274: Removal of Cuboid Bone for Talipes-Equino-Varus, 276; Hemorrhages, 277; Dilation of the Urethra by the Uterine Itself, 278; Electric Light, 279: All of the Metric System Required in Medicine, 280; Treatment of Ganglion. 230; Turpentine as an External Application in Small Pox, 280.

### NOTES, QUERIES AND FORMULÆ.

Capillary Bronchitis, 281: For Intermittent Fever with Congestion of Liver, 281: For Intermittent Fever with Irritable Stomach, 281; For Malarial Dyspepsia, 282; For Diarrhœa, 282; Citrate of Iron, Quinia and Strychnia, 282; Elixir of Calisaya, 282; Gould's Diarrhœa Mixture, 282; Ailanthus Glandulosa in Dysentery, 282; Sulphate of Manganese, 283; The Hypodermic Use of Chloral in Cholera, 283; Elixir of Calisaya and Bismuth, 283: Elixir of Valerinate of Ammonium, 283; Onychia Maligna, 283; Elixir of Quinia and Iron, 283; Cough Syrup, 283.

### SCIENTIFIC ITEMS.

Brute Ceremonies, 284: Velocity of Nerve-Impulses, 284; Hatching Silkworms at will, 284; The New Met 1 Gallium, 284.

### EDITORIAL AND MISCELLANEOUS.

Yellow Fever—New Theory of its Propagation. 285: Professional Taxes, 287; New Departure in Dentistry, 287; Princeton Review, 287; Wood's Library of Standard Medical Authors, 287 Rhinoplastic Operation for Epithelioma, 287; Dr. B. M. Walker's Cure of Umbilical Hernia, 287; Book Notices, 287.

